# Viral Sequence Variation in Chronic Carriers of Hepatitis C Virus Has a Low Impact on Liver Steatosis

**DOI:** 10.1371/journal.pone.0033749

**Published:** 2012-03-29

**Authors:** Marion Depla, Louis d'Alteroche, Amélie Le Gouge, Alain Moreau, Christophe Hourioux, Jean-Christophe Meunier, Julien Gaillard, Anne de Muret, Yannick Bacq, Farhad Kazemi, Aurélie Avargues, Emmanuelle Roch, Eric Piver, Catherine Gaudy-Graffin, Bruno Giraudeau, Philippe Roingeard

**Affiliations:** 1 INSERM U966, Université François Rabelais and CHRU de Tours, Tours, France; 2 Service d'Hépatogastroentérologie, Hôpital Trousseau, CHRU de Tours, Tours, France; 3 INSERM CIC 0202, Université François Rabelais and CHRU de Tours, Tours, France; 4 Unité de Biologie Cellulaire, Hôpital Bretonneau, CHRU de Tours, Tours, France; 5 Plate-Forme RIO des Microscopies, PPF ASB, Université François Rabelais, Tours, France; 6 Service d'Anatomie et Cytologie Pathologiques, Hôpital Trousseau, CHRU de Tours, Tours, France; 7 Service d'Hépatogastroentérologie, Centre Hospitalier de Blois, Blois, France; 8 Service de Biochmie, Hôpital Trousseau, CHRU de Tours, Tours, France; 9 Service de Bactériologie-Virologie, Hôpital Bretonneau, CHRU de Tours, Tours, France; St.Louis University, United States of America

## Abstract

Most clinical studies suggest that the prevalence and severity of liver steatosis are higher in patients infected with hepatitis C virus (HCV) genotype 3 than in patients infected with other genotypes. This may reflect the diversity and specific intrinsic properties of genotype 3 virus proteins. We analyzed the possible association of particular residues of the HCV core and NS5A proteins known to dysregulate lipid metabolism with steatosis severity in the livers of patients chronically infected with HCV. We used transmission electron microscopy to quantify liver steatosis precisely in a group of 27 patients, 12 of whom were infected with a genotype 3 virus, the other 15 being infected with viruses of other genotypes. We determined the area covered by lipid droplets in liver tissues and analyzed the diversity of the core and NS5A regions encoded by the viral variants circulating in these patients. The area covered by lipid droplets did not differ significantly between patients infected with genotype 3 viruses and those infected with other genotypes. The core and NS5A protein sequences of the viral variants circulating in patients with mild or severe steatosis were evenly distributed throughout the phylogenic trees established from all the collected sequences. Thus, individual host factors seem to play a much greater role than viral factors in the development of severe steatosis in patients chronically infected with HCV, including those infected with genotype 3 viruses.

## Introduction

Hepatitis C virus (HCV), which infects 130 million people worldwide, is a major cause of liver disease. It causes liver injury, resulting in chronic hepatitis, fibrosis and, eventually, cirrhosis and hepatocellular carcinoma (HCC). Progression towards end-stage liver disease is influenced by several cofactors including sex (progression more likely in men), age at infection, excessive alcohol consumption, obesity and co-infection with hepatitis B virus (HBV) or human immunodeficiency virus (HIV). Moreover, retrospective studies have reported an association between the presence and severity of steatosis in chronic HCV infection and advanced fibrosis [1], [2]. Steatosis is characterized by hepatic triglyceride deposition and probably depends on host metabolic and viral factors. HCV core protein deregulates a large number of genes involved in lipid metabolism [3]. In cell culture, HCV core protein associates with lipid droplets (LD) and induces the clustering of these lipid storage organelles [4]. This LD clustering which plays an important role in the viral life cycle could be due to a subcellular redistribution of these organelles [5], but the quantification of these clustered LD suggested that *de novo* LD synthesis in infected cells was more likely [4]. The HCV NS5A protein has also recently been shown to modulate various genes involved in lipid metabolism [6], [7], and to accumulate at the LD surface in cell culture [8]. Nonetheless, the molecular mechanisms underlying HCV-induced steatosis remain unclear. The HCV RNA genome displays considerable diversity, both within and between isolates, and three levels of classification have been adopted, grouping viral sequences into six genotypes, several dozen subtypes and intra-isolate variants [9]. Several clinical studies have demonstrated that the prevalence and severity of steatosis are higher in patients infected with genotype 3 HCV than in patients infected with other genotypes [2], [10]–[12], suggesting a possible direct role for viral proteins in triggering steatosis. However, conflicting conclusions have been drawn from *in vitro* studies on the role of specific residues of the HCV-genotype 3 core protein in steatosis [13]–[15]. Differences in the method of LD quantification between studies may explain these discrepancies. In previous investigations on cultured cells producing various wild-type or mutated HCV core proteins, we established an original method for quantifying steatosis by transmission electron microscopy (TEM) [4], [13]. We investigated the impact of HCV diversity on steatosis severity, by applying this method to the analysis of liver biopsy specimens from chronic HCV carriers with viruses of genotype 3 or other genotypes. We aimed to quantify precisely the accumulation of LD in liver tissues. We also determined the sequence of the core and NS5A proteins of the viruses infecting these patients, to evaluate the correlation between specific amino-acid residues or motifs and steatosis severity.

## Materials and Methods

### Ethics Statement

All patients included in this study signed a written informed consent form, in accordance with French regulations. This study was approved by the Institutional Review Board of Tours University Hospital (Comité de Protection des Personnes - CPP).

### Patients

HCV-infected patients meeting the following criteria were recruited from two hospitals in the Loire Valley (Tours, Blois): (i) known chronic HCV infection; (ii) never treated or with antiviral treatment failure resulting in no further treatment for at least 11 months; (iii) known genotype of the infecting virus; (iv) age between 18 and 75 years; (v) need for histological monitoring of the liver, justifying liver biopsy. We excluded patients with metabolic causes of steatosis, by applying the following exclusion criteria: (i) presence of a metabolic syndrome, characterized by a waist ≥94 cm (male)/≥80 cm (female), and at least two of the following criteria: triglyceride concentration >1.7 mmol/l or treated for hypertriglyceridemia, HDL-cholesterol level <1.03 mmol/l (male)/<1.29 mmol/l (female), systolic blood pressure ≥130 mmHg/diastolic blood pressure ≥85 mmHg or treated arterial hypertension, sugar concentration in a fasting blood sample ≥5.6 mmol/l or treated diabetes; (ii) suspected associated hepatic disease (Wilson's diseases, alpha1-antitrypsin deficiency, hemochromatosis, autoimmune hepatitis); (iii) alcohol consumption >2 glasses/day; (iv) HIV or HBV coinfection; (v) “by pass” gastrointestinal gastroplasty, parenteral nutrition, extensive resection of the small intestine, intestinal bacterial overgrowth; (vi) drug treatment potentially inducing or interfering with liver steatosis.

Twenty-seven patients were included: 12 patients infected with HCV genotype 3 and 15 patients infected with another genotype (14 genotype 1 and 1 genotype 5). At the time of liver biopsy, serum viral load (IU/ml) was determined, together with fasting insulin and glucose concentrations, ALAT, ASAT, γGT and alkaline phosphatase levels (IU/l).

### Steatosis quantification in liver biopsy specimens

Liver biopsies were examined blind to patient information by a pathologist (A.d.M), for routine histological diagnosis. A slice (at least 15 mm) of each liver biopsy specimen was fixed in formalin and embedded in paraffin. Sections were cut and stained with hematoxylin-eosin-safran. Fibrosis and activity were graded on the basis of METAVIR scores. For comparison with TEM results, steatosis was also routinely graded 0 (0% hepatocytes with fat), 1 (<20% hepatocytes with fat), 2 (20–50% hepatocytes with fat), or 3 (>50% hepatocytes with fat). The remainder of each liver biopsy specimen (at least 5 mm) was fixed and treated for standard TEM, as previously described [4], [13]. LD in ultrathin sections of these liver biopsy specimens were quantified with a modified version of a method originally developed for use in cultured cells [4], [13]. TEM grids were analyzed blind to patient information. LD diameter was determined on the computer screen for five consecutive squares (5000 µm^2^ each) of two independent TEM grids (i.e. 10 different areas of 5000 µm^2^ of hepatic tissue, for each biopsy). LD diameter was then converted into a disk area in µm^2^. The areas covered by the LD over which a TEM grid bar is partly superimposed were determined specifically with the Image J program (National Institute of Health, USA). By summing these areas, we were able to determine the cumulative LD area in µm^2^ per TEM grid square. For each biopsy, we summed the areas in the 10 squares analyzed.

### Statistical analysis of the cumulative LD areas determined by TEM

Samples from the patients were classified as infected with a genotype 3 virus or with a virus of another genotype. The differences between these groups were assessed with Fisher's exact tests for qualitative variables and Wilcoxon tests for quantitative variables. Cumulative LD areas were compared between the two groups, in a non parametric Wilcoxon test. The correlation between the results obtained by TEM and routine histological diagnosis was assessed by calculating Spearman's rank correlation coefficient with a 95% confidence interval. Data were analyzed with SAS 9.2 and R software.

### RNA extraction, cDNA synthesis, PCR amplification and sequence analysis

RNA was extracted from 140 µl of serum with the QIAamp Viral RNA Mini Kit (Qiagen) and stored at −80°C until processing. For the amplification of core and NS5A coding regions, RT-PCR was performed for 50 min with the Superscript III First Strand Synthesis System (Invitrogen), with 8 µl of the extracted RNA and 2 µM custom primers designed on the basis of information from the HCV sequence database of the Los Alamos National Laboratory (http://hcv.lanl.gov). Amplification was performed with the Platinum PCR Supermix High-Fidelity DNA polymerase (Invitrogen), by simple PCR (core region) or nested PCR (NS5A region), with the custom primers and hybridization temperatures reported in the supporting Table S1.

Five to 18 clones were generated per patient for each region (mean: 9 for core and 13 for NS5A). Core and NS5A quasispecies variant sequences were determined on an ABI PRISM 3100 machine (Applied Biosystems). BioEdit software (http://www.mbio.ncsu.edu/) was used to compare amino-acid sequences. Phylogenetic and molecular evolutionary analyses were conducted with MEGA 5 software [16], by the maximum likelihood method. Using the substitution rate for each amino acid over time, we estimated the likelihood (bootstrap value >75%) of the position and length of the branches of the tree. An evolutionary distance of 0.01 corresponded to 10 mutations per 1000 residues.

We specifically assessed the association of each individual residue from all core and NS5A protein sequences with the presence or absence of steatosis, using the Los Alamos – NCBI Database (Highlighter tool: http://hcv.lanl.gov/content/sequence/HIGHLIGHT/highlighter.html) and CLC Main Workbench 6 software.

## Results

### General characteristics of patients at the time of liver biopsy

Patients were assigned to one of the two groups on the basis of viral genotype (3 or non-3). These two groups did not differ in terms of sex ratio, age, BMI, duration of infection, source of infection, alcohol intake, ALAT, ASAT and alkaline phosphatase concentrations, glycemia, viral load or METAVIR score (Table 1). However, patients with genotype 3 viruses had lower serum cholesterol, TG and γGT concentrations than patients with viruses of other genotypes. The difference in serum cholesterol concentration between patients infected with genotype 3 viruses and those infected with viruses of other genotypes has been reported elsewhere [10], [17], but the impact of HCV genotype on TG level remains unclear. Our inclusion criteria were designed to exclude patients with a metabolic syndrome, but we nevertheless carried out homeostasis model assessment of insulin resistance (HOMA-IR) [18] with the measurement of fasting insulin and glucose concentrations at the time of liver biopsy. Median HOMA-IR score was slightly higher in patients infected with non-3 genotypes, but this difference was not significant (Table 1). However, four patients with non genotype 3 viruses had a HOMA-IR score >4, suggesting possible insulin resistance in these patients (Table 1).

**Table 1:** Demographic, biochemical and histological characteristics of the patients at the time of liver biopsy.

	Genotype 3^a^	Genotype non 3	p-value
	*n = 12*	*n = 15*	
Male (%)	8 (66.7)	8 (53.3)	NS
Ethnicity			NS
European Caucasian	12 (100.0)	14 (93.3)	
North-African Caucasian	0 (0.0)	1 (6.7)	
Age (median)	49.0 [47.0; 50.5]	51.0 [48.0; 61.0]	NS
BMI (median)	22.9 [21.2; 24.8]	24.7 [21.4; 26.1]	NS
Duration of infection (median)	28.5 [20.5; 31.5]^b^	29.0 [24.0; 31.0]^c^	NS
Source of infection (%)			NS
Drug addiction	6 (50.0)	5 (33.3)	
Transfusion	2 (16.7)	6 (40.0)	
Other	2 (16.7)	2 (13.3)	
Unknown	2 (16.7)	2 (13.3)	
Alcohol intake (%)			NS
0 glass/day	10 (83.3)	11 (73.3)	
1–2 glass/day	2 (16.7)	4 (26.6)	
Biochemical data (median)			
ALAT (IU/l)	120.0 [49.5; 144.5]	79.0 [54.0; 111.0]	NS
ASAT (IU/l)	71.0 [40.5; 92.5]	54.0 [40.0; 94.0]	NS
GGT (IU/l)	71.5 [43.0; 103.5]	130.0 [99.0; 233.0]	0.012
Alkaline phosphatase	69.5 [61.0; 95.5]	81.0 [64.0; 108.0]	NS
Total cholesterol (mmol/l)	3.9 [3.3; 4.0]	4.8 [4.4; 5.7]	0.0002
Glycemia (mmol/l)	5.0 [4.7; 5.6]	5.4 [4.8; 5.8]	NS
Triglyceridemia (mmol/l)	0.74 [0.62; 1.13]	1.36 [1.14; 1.67]^d^	0.004
HOMA-IR score (median)	1.40 [0.88; 2.51]	1.98 [1.36; 4.29]^d^	NS
Insulin resistance (HOMA-IR >4) (%)	0 (0.0)	4 (26.6)	NS
Viral load (log IU/ml)	6.1 [5.8; 6.5]	6.2 [5.8; 6.6]	NS
METAVIR Score (%)			NS
A1F1	3 (25.0)	3 (20.0)	
A1F2	1 (8.3)	1 (6.7)	
A1F4	1 (8.3)	0 (0.0)	
A2F1	2 (16.7)	3 (20.0)	
A2F2	1 (8.3)	3 (20.0)	
A2F3	3 (25.0)	3 (20.0)	
A2F4	0 (0.0)	2 (13.3)	
A3F4	1 (8.3)	0 (0.0)	

a Determined with the Versant HCV Genotype Assay.

b Data missing for 4 patients.

c Data missing for 2 patients.

d Data missing for 1 patient.

### Steatosis quantification by TEM and analysis

**Figure 1:**
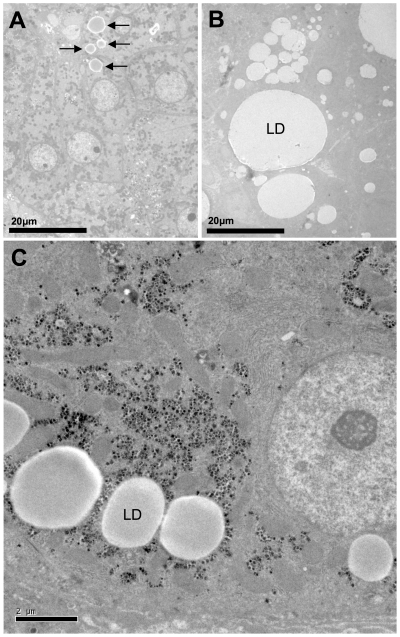
TEM observation of representative liver biopsy specimens from three patients chronically infected with HCV. The liver in A (patient P16) shows a small number of small LD (arrows) and was found to have a lipid droplet area <1000 µm^2^ per 50,000 µm^2^ of tissue. The liver in B (patient P20) has extremely large LD and was found to have a LD area >10,000 µm^2^ per 50,000 µm^2^ of tissue. The TEM photograph in C shows a high magnification of an individual hepatocyte with several large LD, taken from a liver biopsy specimen (patient P03) considered intermediate to the other two in terms of steatosis severity. Scale bars: 20 µm in A and B; 2 µm in C.

**Figure 2:**
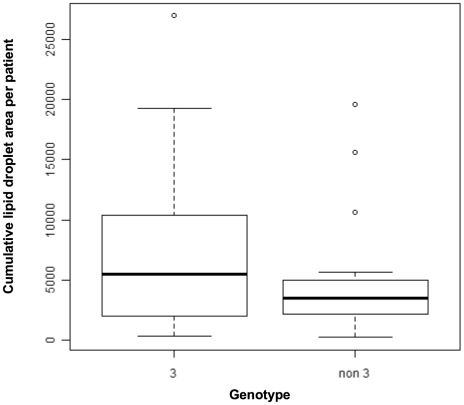
Comparative analysis of the cumulative LD areas determined by TEM on liver biopsy specimens from patients infected with genotype 3 viruses and viruses of other genotypes, in µm^2^. Cumulative LD areas were summed for all patients with genotype 3 infection (left boxplot) and for patients infected with viruses of other genotypes (right boxplot). Boxplots show the median LD area (horizontal black line), the 25^th^ and the 75^th^ percentile. Dot plots indicate the extreme values of cumulative LD area. There was no significant difference between the two groups (5461.1[2165.2; 10027.9] for the genotype 3 group versus 3538.7[2184.6; 4984.4] for the non-3 group, p = 0.581).

The area covered by LD in 10 different areas of 5000 µm^2^ of hepatic tissue (50,000 µm^2^ in total) was determined by TEM for each liver biopsy specimen. Figure 1 shows LD on ultrathin sections from the liver biopsy specimens of three different patients with representative profiles. A box plot was generated for the analysis of these cumulative LD areas as a function of viral genotype (Fig. 2). Median cumulative LD area did not differ significantly between the two groups (see the horizontal dark line in each box): 5461.1[2165.2; 10027.9] for the genotype 3 group, versus 3538.7[2184.6; 4984.4] for the genotype non-3 group (p = 0.581). However, the cumulative LD areas of patients infected with genotype 3 viruses were much more variable than those of patients infected with viruses of other genotypes (Fig. 2). The quantification of LD by TEM was compared with the results of steatosis assessment during routine histological analysis of the liver biopsy specimen. With a Spearman's rank correlation coefficient of 0.87 [0.73; 0.94], histological steatosis grade was correlated with the cumulative LD area obtained by TEM. The slight differences observed may result from the detection, by TEM, of the accumulation of small LD (microsteatosis) in some patients considered free of steatosis in routine histological assessments. All four patients infected with non genotype 3 viruses displaying suspected insulin resistance (HOMA-IR >4 ; patients 7, 8, 15 and 18) had mild steatosis.

### Analysis of HCV core and NS5A protein sequences as a function of steatosis severity

**Figure 3:**
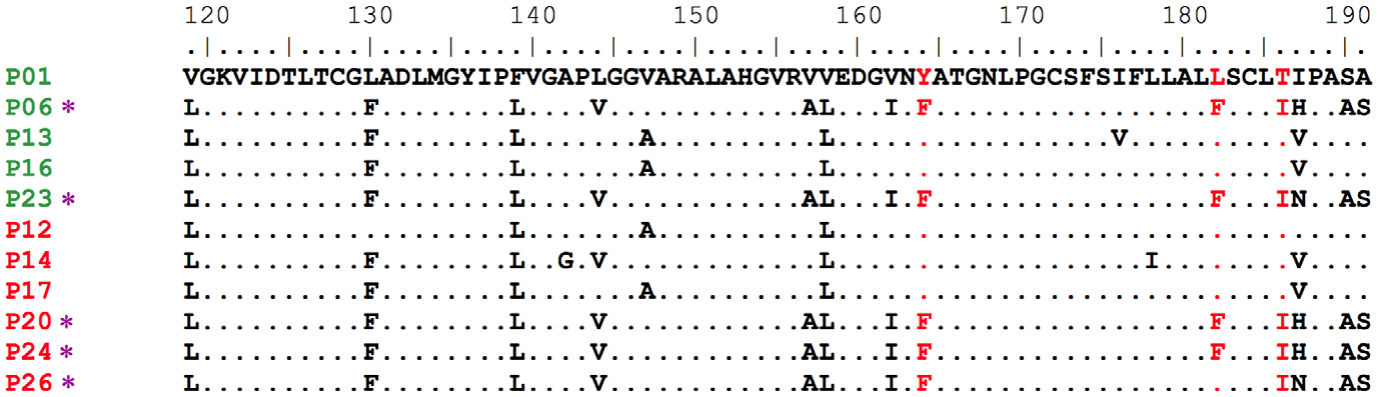
Alignment of amino acids 119–191 of the core protein consensus sequences from 5 patients with little or no steatosis (in green) and 6 patients with severe steatosis (in red). The 5 patients in with little or no steatosis had a LD area <1000 µm^2^ per 50,000 µm^2^ of liver tissue. The 6 patients with severe steatosis had a LD area >10,000 µm^2^ per 50,000 µm^2^ of tissue. Patients infected with genotype 3 strains are indicated with an asterisk (*) and such patients are present in both groups. The residues at each position are indicated with the single-letter amino-acid code. Amino acids identical to the first sequence are indicated by a dot. The positions of the residues shown in previous studies to increase LD accumulation *in vitro*, in cultured cells, are indicated in red. No association was found between a particular amino-acid residue or motif and the presence of severe steatosis.

We first investigated the involvement of HCV core and NS5A protein residues in steatosis severity, in an analysis on a restricted number of patients: those with severe liver steatosis, with an LD area greater than 10,000 µm^2^ on TEM (6 patients: 3 with a genotype 3 virus, 2 with a genotype 1 virus and 1 with a genotype 5 virus); and those with very little or no steatosis, characterized by an LD area less than 1,000 µm^2^ on TEM (5 patients: 3 with a genotype 1 virus and 2 with a genotype 3 virus). No association was found between particular amino-acid residues or motifs anywhere in the core region (domains 1, 2 and 3) and severe steatosis (Supporting Fig. S1). For example, Figure 3 shows the alignment of HCV core protein domains 2 (D2) and 3 (D3) of the consensus sequence determined for the 11 patients. HCV core protein D2 (residues 119–173) and D3 (residues 174–191) have been implicated in the association of the protein with the surface of the LD, and some key residues have been shown, *in vitro*, to increase LD accumulation in cultured cells [13], [15]. Residues highly specific to genotype 3 strains, such as the phenylalanine residue in position 164, were found in all genotype 3 viruses, but the presence of this particular residue was not associated with steatosis severity (Fig. 3 and Supporting Fig. S1), despite the previous implication of this residue in severe steatosis *in vitro* [13]. Similarly, the 182LI186 and 182FV186 motifs, which have also been associated with higher levels of LD accumulation in *in vitro* models [15], were not associated with severe steatosis in our patients (Fig. 3). Indeed, only two patients were infected with viruses bearing the 182LI186 or 182FV186 sequences (patients 26 and 27, Supporting Fig. S1), but only one of these patients had severe steatosis. Patient 26 was the only one of the six patients with severe steatosis found to be infected with a virus bearing the 182FV186 or 182LI186 sequence.

**Figure 4:**
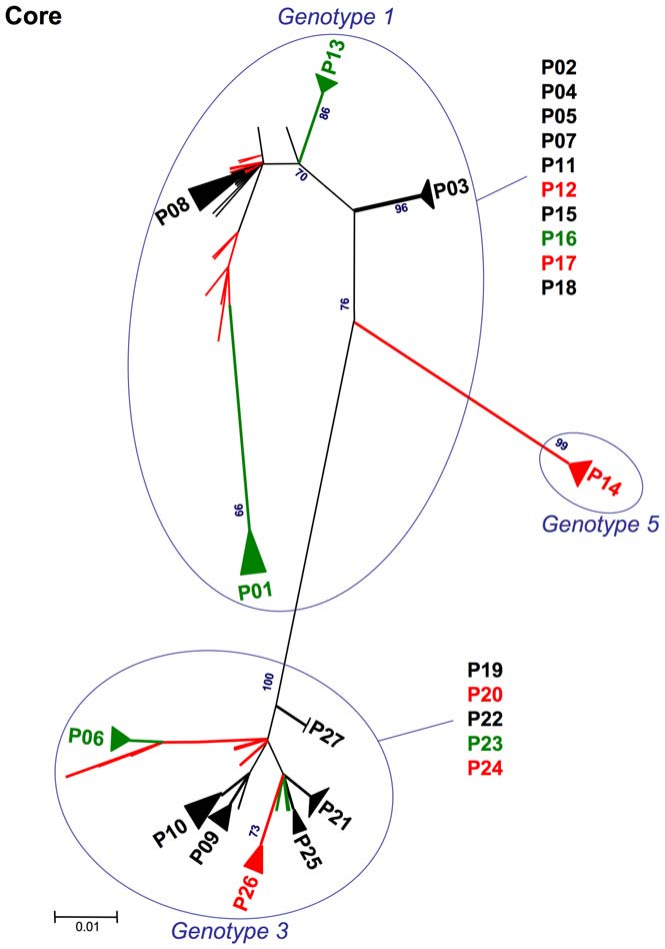
Unrooted phylogenic trees for the core protein sequences of all variants identified in the 27 patients included in the study. The 5 patients in green had a LD area <1000 µm^2^ per 50,000 µm^2^ of liver tissue. The 6 patients in red had a LD area >10,000 µm^2^ per 50,000 µm^2^ of tissue. The remaining 16 patients, with steatosis of intermediate severity, are shown in black. Diversity in individual patients is represented by a triangle at the top of the branches, with the magnitude of the diversity observed increasing with the area of the triange. Bootstrap values exceeding 75% are shown (numbers in dark blue). Scale bars indicate evolutionary distance (0.01 indicates 10 mutations for every 1000 residues). Patients are grouped according to viral genotype. Patients with little or no steatosis (green) and those with severe (red) steatosis are evenly distributed over these phylogenic trees. No clustering according to steatosis status was observed.

We then established a phylogenetic tree from the core protein sequences of all variants found in all patients. The sequences clustered by genotype (Fig. 4), with one group for genotype 1 sequences (14 patients), one group for genotype 3 sequences (12 patients) and an isolated sequence for genotype 5 (1 patient). Due to the high degree of core sequence conservation within the various genotypes, clones from the same patient did not necessarily cluster, instead mixing with those from other patients. Thus, clusters were found for only twelve patients, for whom diversity within the various clones is indicated by the triangle at the top of the branch. Patients with little or no (green) and severe (red) steatosis were evenly distributed over this phylogenic tree, with no particular cluster of the phylogenic tree associated with severe steatosis.

**Figure 5:**
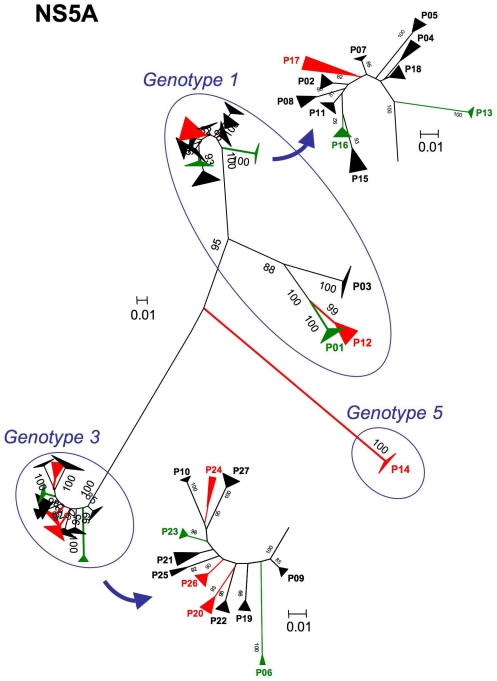
Unrooted phylogenic trees for the NS5A protein sequences of all variants identified in the 27 patients included in the study. The 5 patients in green had a LD area <1000 µm^2^ per 50,000 µm^2^ of liver tissue. The 6 patients in red had a LD area >10,000 µm^2^ per 50,000 µm^2^ of tissue. The remaining 16 patients, with steatosis of intermediate severity, are shown in black. Diversity in individual patients is represented by a triangle at the top of the branches, with the magnitude of the diversity observed increasing with the area of the triange. Bootstrap values exceeding 75% are shown (numbers in dark blue). Scale bars indicate evolutionary distance (0.01 indicates 10 mutations for every 1000 residues). Patients are grouped according to viral genotype. Patients with little or no steatosis (green) and those with severe (red) steatosis are evenly distributed over these phylogenic trees. No clustering according to steatosis status was observed.

The NS5A protein sequences of the variants in a given patient were highly heterogeneous and we were unable to identify a consensus sequence for most of the patients. We then constructed phylogenetic trees with all these sequences. As for the core protein, the distribution of variants between the three groups depended on genotype (Fig. 5). NS5A protein sequences were highly heterogeneous and we were able to evaluate diversity for each patient (triangles at the end of each branch). However, patients with little or no steatosis (green) and those with severe (red) steatosis were again evenly distributed over the entire tree, indicating a lack of association between any particular cluster of the phylogenic tree and severe steatosis.

**Figure 6:**
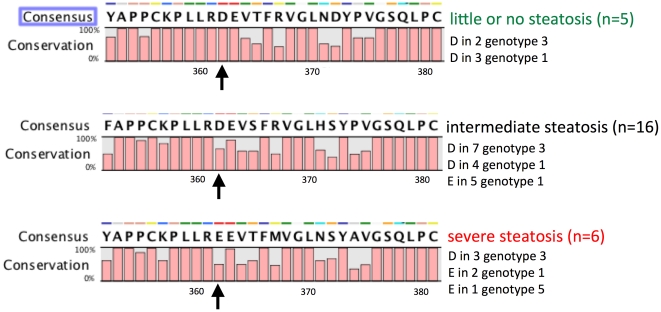
Conservation of amino acids 161–181 of the NS5A protein sequences of all variants identified in the 27 patients included in the study. The sequences were classified into three different groups : (i) patients with little or no steatosis, with a LD area <1000 µm^2^ per 50,000 µm^2^ of liver tissue (n = 5) ; (ii) patients with severe steatosis, with a LD area >10,000 µm^2^ per 50,000 µm^2^ of tissue (n = 6) ; (iii) patients with a steatosis of intermediate severity (n = 16). Using CLC Main Workbench 6 software, we determined, for each group, the most frequent residue in each position and the percentage conservation of these residues. The D in position 362 in the core-NS5A fused sequence (arrow, residue 171 in the NS5A protein) was highly conserved in all genotype 3 viruses from all groups. For the other genotypes, the D on position 362/171 was characteristic of the variants found in patients with little or no steatosis, whereas an E in the same position was characteristic of the variants found in patients with severe steatosis.

The binding of the core protein to NS5A has been shown to be critical for virus particle assembly [19]. This interaction may potentiate the effects of these proteins in lipid accumulation. We therefore investigated whether any particular association of residues between the core and NS5A sequences was correlated with steatosis severity. We then established a new phylogenic tree from the core-NS5A fused sequences. For each patient, we fused the most frequent HCV core sequence (highly conserved among the variants for a given patient) with each of the NS5A variant sequences. In this new analysis (Supplemental Fig. S2), the branches of the phylogenic tree were positioned similarly to those in the analysis of the NS5A protein alone, the sequences associated with little or no steatosis (green) and those associated with severe (red) steatosis being evenly distributed over the entire tree. This suggests that there is no association between core and NS5A residues to form particular clusters correlated with severe steatosis. A serine cluster (serines in position 432, 434 and 437 in the genotype 1a H77 sequence) in the C-terminal part of the NS5A protein has been shown to be determinant for the NS5A-core interaction [19]. We therefore focused particularly on these residues in our variant sequences. However, this serine cluster in position 432/434/437 was highly conserved in the NS5A sequences from all variants in all patients (data not shown). We then investigated the potential association of each individual residue of the core and NS5A proteins with the presence or absence of steatosis. We overcame the problem of genotype specificity by first analyzing separately the patients infected with genotype 1 viruses (n = 14) and genotype 3 viruses (n = 12). The core and NS5A protein sequences of all variants were compared with the NCBI consensus sequence for genotype 1 (supplemental Fig. S3) or genotype 3 (supplemental Fig. S4) viruses. No residue or group of residues was found to be associated with severe steatosis, in either of the groups. However, this analysis led to the identification of a residue in position 362 in the core-NS5A fused sequences that was characteristic of the genotype 1 variants found in patients with little or no steatosis (arrow in Fig. S3). We then assigned the variant sequences from the 27 patients to three groups (patients with little or no steatosis, patients with intermediate steatosis, and patients with severe steatosis). Using CLC Main Workbench 6 software, we determined, for each group, the most frequent residue in each position and determined the percentage conservation of these residues, focusing in particular on the residue in position 362 (aa 171 in the NS5A protein of the H77 strain). The D residue in 362 was highly conserved in all genotype 3 viruses, regardless of the presence or absence of steatosis (Fig. 6). However, in the other genotypes, the D in position 362 was characteristic of the variants found in patients with little or no steatosis, whereas an E residue in position 362 was typically found in the variants from patients with severe steatosis (Fig. 6). However, the number of patients with non genotype 3 viruses studied was too small to draw any firm conclusion about the role of this residue in virus-induced steatosis.

## Discussion

The mechanisms leading to liver steatosis in chronic hepatitis C remain unclear, probably due to the existence of two different entities: (i) steatosis with common causes, such as obesity, diabetes mellitus or alcohol intake, (ii) steatosis without known risk factors, presumably at least partly related to viral infection. Steatosis seems to be mostly (but probably not exclusively) virus-induced in infections with genotype 3 viruses, whereas it is principally associated with host factors in infections with viruses of other genotypes. We investigated this issue by an approach allowing the precise quantification of steatosis in the liver tissues of patients infected with viruses of genotype 3 and of other genotypes. This study included only a small number of patients due to (i) inclusion being limited to patients requiring histological monitoring of the liver justifying liver biopsy, (ii) the difficulty of extending the meticulous and time-consuming quantification of LD by TEM to a large number of patients. In formalin-fixed tissues embedded in paraffin for sectioning for routine histological diagnosis, the LD fuse and tend to form holes, deformed to various extents [20], and steatosis is evaluated by determining the percentage of hepatocytes presenting this pattern. This routine evaluation of steatosis was carried out for comparison with our original TEM method, initially developed for a more precise quantification of steatosis in liver biopsy fragments. This small number of patients may have limited our analysis, but the main advantage of TEM was the precise determination of LD area in liver tissues that it allowed, including the quantification of small LD (microsteatosis) in some parts of the tissue considered steatosis-free on routine histological analysis. However, we are aware of the limitations associated with the study of small fragments of biopsy specimens by TEM as opposed to larger tissue sections by conventional histology. The overall correlation between the results obtained with the two approaches was good. Using this original technique, we showed that the cumulative area covered by LD in the livers of patients infected with genotype 3 viruses did not differ significantly from that in the livers of patients infected with viruses of other genotypes. These results conflict with those of other studies reporting a higher frequency of severe steatosis in patients infected with genotype 3 viruses [2], [10]–[12], but are consistent with several studies showing this link to be non significant [21], [22]. Serum cholesterol concentration was lower in patients infected with genotype 3 viruses than in patients infected with other genotypes, as reported in other studies [10], [12], [17], [22], and this difference could not be accounted for by the demographic profiles of the patients. Lower serum cholesterol concentrations have been found in patients infected with various HCV genotypes, probably due to a global deficit in lipid and lipoprotein metabolism induced by HCV that might be exacerbated in patients infected with genotype 3 viruses [23]. However, this lower serum cholesterol concentration in patients infected with genotype 3 viruses was not associated with more severe steatosis in our study.

The mechanisms by which viral proteins affect the development of genotype-3-specific steatosis remain hypothetical. The HCV core protein accumulates at the surface of LD *in vitro* and *in vivo*, induces steatosis in the livers of transgenic mice and deregulates a large number of genes involved in lipid metabolism [3]. It has therefore been extensively studied. Several *in vitro* studies have shown a specific interaction between the genotype 3 core protein and lipid metabolism. Early experiments based on the production of HCV core proteins from viruses of different genotypes showed that all core proteins induced triglyceride accumulation, but that this phenomenon was strongest with genotype 3 core protein [24]. This protein strongly induces fatty acid synthase (FAS), an enzyme required for intracellular triglyceride production, in a sterol response element binding protein 1 (SREBP1)–dependent manner [25]. HCV core protein induces the cleavage of sterol regulatory element binding proteins and their phosphorylation, by triggering oxidative stress, leading to fatty acid synthesis, this this phenomenon is generally more efficient with a genotype 3 core protein [26]. The phosphatase and tensin homolog (PTEN) has recently been shown to be downregulated by a genotype 3 core protein, via a mechanism involving a microRNA-dependent blockade of PTEN mRNA translation, whereas genotype 1 core protein has no effect [27]. The functional differences between genotype 1 and 3 core proteins probably reflect differences in amino-acid sequences, although these sequences are very similar. However, single amino-acid differences between these proteins may modulate local folds, stability and interactions with host cell proteins. Core protein domain 2 (amino acids 119–173) has been implicated in LD association [3], [13]. Within this domain, the Y164F mutation of a genotype 1 core protein, resulting in the incorporation of a residue highly specific to genotype 3 strains, has been shown to increase LD accumulation *in vitro*, in cellular models [13]. This particular mutant has also been implicated in the stronger FAS activation observed with the genotype 3 core protein [25]. One group has also reported a possible link between polymorphism in domain 3 (amino acids 174–191) of the genotype 3 core protein and steatosis [15], [28]. The FV or LI combination of residues at positions 182 and 186 in viral sequences (as opposed to FI) has been reported to be associated with the presence of severe intrahepatic steatosis in patients. The *in vitro* production of core proteins with these steatosis-associated polymorphisms increases intracellular lipid levels over those obtained with core proteins from isolates not associated with steatosis [15], [28]. However, the presence of these particular residues was not correlated with severe steatosis in our patients. We identified no particular amino-acid residues or motifs associated with severe steatosis within core sequences. Furthermore, careful phylogenic analysis of all the core sequences collected showed an absence of sequence clustering as a function of steatosis status. These results are consistent with those of Piodi *et al*. [14], who found no difference between the genes encoding genotype 3 virus core proteins between patients with and without steatosis. However, as Piodi *et al.* showed that the genotype 3 core protein induced the formation of larger LD *in vitro* than the genotype 1 core protein [14], we also analyzed individual LD diameter and area on liver biopsy specimens. Again, we found no significant difference between genotype 3 and the other genotypes (p = 0.92; data not shown).

HCV NS5A protein colocalizes with the core protein on LD and interacts with apolipoprotein (Apo) A1, ApoB and ApoE [8] and with peroxisome proliferator-activated receptor gamma (PPAR-gamma) [7]. Furthermore, the NS5A protein has recently been shown to induce steatosis in a transgenic mouse model [6]. We therefore analyzed the NS5A sequences of the multiple variants circulating in our patients. The NS5A protein sequences of the variants in a given patient were highly heterogeneous, and we were therefore unable to identify a consensus sequence for most of the patients for sequence alignment analysis. Phylogenic analysis of all the N5A sequences collected showed no clustering according to steatosis status. Finally, when analyzing NS5A sequence conservation by genotype, we showed that residue 171 of the NS5A protein was potentially associated with steatosis status, but only in infections with genotypes other than genotype 3 (D in patients with little or no steatosis, E in patients with severe steatosis). However, these two amino acids have similar properties and too few patients were studied to draw any firm conclusions on the role of this specific residue in the steatosis induced by non genotype 3 viruses.

Thus, our study suggests that core and NS5A protein variability in HCV genotypes and strains has little effect on steatosis severity in chronic HCV carriers. In particular, the effects of the genotype 3 core protein observed *in vitro* [13], [15], [24], [25], [27], [28] seemed to be much milder *in vivo*, in conditions in which host factors may have a much stronger effect on steatosis severity. This is consistent with the absence of a direct relationship *in vivo* between steatosis and the presence of HCV proteins within steatotic hepatocytes [22]. Furthermore, the specific induction of higher SREBP1 levels by a genotype 3 core protein *in vitro* [25] was not confirmed in a recent analysis of gene expression in the livers of human patients infected with HCV of various genotypes [29]. Our findings are consistent with those of Piodi *et al.* [14], suggesting that host factors play a greater role in the development of severe liver steatosis than viral factors in patients infected with a genotype 3 virus, contrary to the conclusions of the clinical studies previously addressing this question. Interestingly, recent data have suggested that the polymorphism of various genes, including the PPAR-gamma, IL-28B, adiponutrin and microsomal triglyceride transfer protein (MTP) genes, may influence the development of more severe steatosis in chronic carriers of HCV [30]–[34]. An indirect factor, such as alcohol consumption, which some patients might find it difficult to acknowledge, may also have contributed to the difference reported in previous studies between patients infected with genotype 3 viruses and viruses of other genotypes. Indeed, epidemiological studies in Western Europe, including France, have shown that alcohol abuse is frequently observed in patients with a history of intravenous drug use (IVDU) and that genotype 3 infection is frequent in patients with a history of IVDU [35].

In conclusion, our study suggests that individual host factors seem to play a greater role than viral factors in the development of severe steatosis in patients chronically infected with HCV, including those infected with genotype 3 viruses.

## Supporting Information

Figure S1 Alignment of the whole core protein consensus sequences from the viruses identified in the 27 patients included in the study. The 5 patients in green had a LD area <1000 µm^2^ per 50,000 µm^2^ of liver tissue. The 6 patients in red had a LD area >10,000 µm^2^ per 50,000 µm^2^ of tissue. The remaining 16 patients, with steatosis of intermediate severity, are shown in black. Patients infected with genotype 3 strains are indicated by an asterisk (*).
(EPS)

Figure S2 Unrooted phylogenic trees for the core-NS5A fused sequences of the variants identified in the 27 patients included in the study. For each patient, we fused the most frequent HCV core sequence (highly conserved among the variants for a given patient) with each of the NS5A variant sequences. Patients with little or no steatosis (green) and those with severe (red) steatosis are evenly distributed over these phylogenic trees.
(EPS)

Figure S3 Comparison of the core-NS5A fused sequences of the variants identified in the 14 patients infected with a genotype 1 virus with the NCBI consensus sequence for genotype 1. Patients with little or no steatosis appear in green and patients with severe steatosis appear in red.
(EPS)

Figure S4 Comparison of the core-NS5A fused sequences of the variants identified in the 12 patients infected with a genotype 3 virus with the NCBI consensus sequence for genotype 3. Patients with little or no steatosis appear in green and patients with severe steatosis appear in red.
(EPS)

Table S1 Sequences of the primers used and conditions for RT-PCR amplification of the core and NS5A protein-coding regions.
(DOC)

## References

[1] Hourigan LF, Macdonald GA, Purdie D, Whitehall VH, Shorthouse C (1999). Fibrosis in chronic hepatitis C correlates significantly with body mass index and steatosis.. Hepatology.

[2] Adinolfi LE, Gambardella M, Andreana A, Tripodi MF, Utili R, Ruggiero G (2001). Steatosis accelerates the progression of liver damage of chronic hepatitis C patients and correlates with specific HCV genotype and visceral obesity.. Hepatology.

[3] Roingeard P, Hourioux H (2008). Hepatitis C virus core protein, lipid droplets and steatosis.. J Viral Hepat.

[4] Depla M, Uzbekov R, Hourioux C, Blanchard E, Le Gouge A (2010). Ultrastructural and quantitative analysis of the lipid droplet clustering induced by hepatitis C virus core protein.. Cell Mol Life Sci.

[5] Boulant S, Douglas MW, Moody L, Budkowska A, Targett-Adams P, McLauchlan J (2008). Hepatitis C virus core protein induces lipid droplet redistribution in a microtubule and dynein dependent manner.. Traffic.

[6] Wang A-G, Lee D-S, Moon H-B, Kim JH, Cho KH (2009). Non-structural 5A protein of hepatitis C virus induces a range of liver pathology in transgenic mice.. J Pathol.

[7] Kim K, Kim KH, Ha E, Park JY, Sakamoto N, Cheong J (2009). Hepatitis C virus NS5A protein increases hepatic lipid accumulation via induction of activation and expression of PPARgamma.. FEBS Let.

[8] Benga WJ, Krieger SE, Dimitrova M, Zeisel MB, Parnot M (2010). Apolipoprotein E interacts with hepatitis C virus nonstructural protein 5A and determines assembly of infectious particles.. Hepatology.

[9] Simmonds P, Bukh J, Combet C, Deléage G, Enomoto N (2005). Consensus proposals for a unified system of nomenclature of hepatitis C virus genotypes.. Hepatology.

[10] Poynard T, Ratziu V, McHutchison J, Manns M, Goodman Z (2003). Effect of treatment with peginterferon or interferon alfa-2b and ribavirin on steatosis in patients infected with hepatitis C.. Hepatology.

[11] Rubbia-Brandt L, Quadri R, Abid K, Giostra E, Malé PJ (2000). Hepatocyte steatosis is a cytopathic effect of hepatitis C virus genotype 3.. J Hepatol.

[12] Hofer H, Bankl HC, Wrba F, Steindl-Munda P, Peck-Radosavjevic M (2002). Hepatocellular fat accumulation and low serum cholesterol in patients infected with HCV-3a.. Am J Gastroenterol.

[13] Hourioux C, Patient R, Morin A, Blanchard E, Moreau A (2007). The genotype 3-specific hepatitis C virus core protein residue phenylalanine 164 increases steatosis in an in vitro cellular model.. Gut.

[14] Piodi A, Chouteau P, Lerat H, Hézode C, Pawlotsky JM (2008). Morphological changes in intracellular lipid droplets induced by different hepatitis C virus genotype core sequences and relationship with steatosis.. Hepatology.

[15] Jhaveri R, McHutchison J, Patel K, Qiang G, Diehl AM (2008). Specific polymorphisms in hepatitis C virus genotype 3 core protein associated with intracellular lipid accumulation.. J Infect Dis.

[16] Tamura K, Peterson D, Peterson N, Stecher G, Nei M, Kumar S (2011). MEGA5: Molecular evolutionary genetics analysis using maximum likelihood, evolutionary distance, and maximum parsimony methods.. Mol Biol Evol.

[17] Siagris D, Christofidou M, Theocharis GJ, Pagoni N, Papadimitriou C (2006). Serum lipid pattern in chronic hepatitis C: histological and virological correlations.. J Viral Hepat.

[18] Eslam M, Kawaguchi T, Del Campo AD, Sata M, Abo-Elneen Khattab M (2011). Use of HOMA-IR in hepatitis C.. J Viral Hepat.

[19] Masaki T, Suzuki R, Murakami K, Aizaki H, Ishii K (2008). Interaction of hepatitis C virus nonstructural protein 5A with core protein is critical for the production of infectious virus particles.. J Virol.

[20] Fujimoto T, Ohsaki Y, Cheng J, Suzuki M, Shinohara Y (2008). Lipid droplets: a classic organelle with new outfits.. Histochem Cell Biol.

[21] Kumar D, Farrell GC, Fung C, George J (2002). Hepatitis C virus genotype 3 is cytopathic to hepatocytes: Reversal of hepatic steatosis after sustained therapeutic response.. Hepatology.

[22] Grassi A, Ballardini G, Susca M, Bianchini F, Bonoli S (2005). HCV liver infection and liver steatosis: evidence for indirect mechanisms in genotype 3?. Aliment Pharmacol Ther.

[23] Piver E, Roingeard P, Pagès J-C (2010). The cell biology of hepatitis C virus (HCV) lipid addiction: molecular mechanisms and its potential importance in the clinic.. Int J Biochem Cell Biol.

[24] Abid K, Pazienza V, de Gottardi A, Rubbia-Brandt L, Conne B (2005). An in vitro model of hepatitis C virus genotype 3a-associated triglyceride accumulation.. J Hepatol.

[25] Jackel-Cram C, Babiuk LA, Liu Q (2007). Up-regulation of fatty acid synthase promoter by hepatitis C virus core protein: genotype-3a core has a stronger effect than genotype-1b core.. J Hepatol.

[26] Waris G, Felmlee DJ, Negro F, Siddiqui A (2007). Hepatitis C virus induces proteolytic cleavage of sterol regulatory element binding proteins and stimulates their phosphorylation via oxidative stress.. J Virol.

[27] Clément S, Peyrou M, Sanchez-Pareja A, Bourgouin L, Ramadori P (2011). Downregulation of PTEN and IRS-1 by HCV 3a core protein triggers the formation of large lipid droplets in hepatocytes.. Hepatology.

[28] Jhaveri R, Qiang G, Diehl AM (2009). Domain 3 of hepatitis C virus core protein is sufficient for intracellular lipid accumulation.. J Infect Dis.

[29] Ryan MC, Desmond PV, Slavin JL, Congiu M (2011). Expression of genes involved in lipogenesis is not increased in patients with HCV genotype 3 in human liver.. J Viral Hepat.

[30] Cai T, Dufour J-F, Muellhaupt B, Gerlach T, Heim M (2011). Viral genotype-specific role of PNPLA3, PPARG, MTTP, and IL28B in hepatitis C virus-associated steatosis.. J Hepatol.

[31] Sookoian S, Castaño GO, Burgueño AL, Gianotti TF, Rosselli MS, Pirola CJ (2009). A nonsynonymous gene variant in the adiponutrin gene is associated with nonalcoholic fatty liver disease severity.. J Lipid Res.

[32] Sookoian S, Pirola CJ (2011). Meta-analysis of the influence of I148M variant of patatin-like phospholipase domain containing 3 gene (PNPLA3) on the susceptibility and histological severity of nonalcoholic fatty liver disease.. Hepatology.

[33] Mirandola S, Osterreicher CH, Marcolongo M, Datz C, Aigner E (2009). Microsomal triglyceride transfer protein polymorphism (-493G/T) is associated with hepatic steatosis in patients with chronic hepatitis C.. Liver Int.

[34] Zampino R, Ingrosso D, Durante-Mangoni E, Capasso R, Tripodi MF (2008). Microsomal triglyceride transfer protein (MTP) −493G/T gene polymorphism contributes to fat liver accumulation in HCV genotype 3 infected patients.. J Viral Hepat.

[35] Pawlotsky JM, Tsakiris L, Roudot-Thoraval F, Pellet C, Stuyver L (1995). Relationship between hepatitis C virus genotypes and sources of infection in patients with chronic hepatitis C.. J Infect Dis.

