# Supplementary material for: Viral Sequence Variation in Chronic Carriers of Hepatitis C Virus Has a Low Impact on Liver Steatosis
Source: PLoS One. 2012 Mar 29;7(3):e33749. doi: 10.1371/journal.pone.0033749 (PMC3315576; doi:10.1371/journal.pone.0033749)
Supplement: Table S1 — Sequences of the primers used and conditions for RT-PCR amplification of the core and NS5A protein-coding regions. (DOC) [file pone.0033749.s005.doc]

**Supporting Table S1.** Sequences of the primers used and conditions for RT-PCR amplification of the core and NS5A protein-coding regions

***___________________________________________________________________________***

| **Primer** | **Sequence** |
| --- | --- |
| CORE sense | 5' CGG ATC CCT TGT GGT ACT GCC TGA TAG GG 3' |
| CORE Gen 1/5 antisense | 5' GGG ATC CTT AGB GAC CAG TTC ATC ATC ATA TCC CA 3' |
| CORE Gen 3 antisense | 5' AGG ATC CTT ACC AAT TCA TCA TCA TAT CCC AAG C 3' |
| NS5A Gen 1 out sense | 5' CAG TGG ATG AAC CGG CTR ATA 3' |
| NS5A Gen 1 out antisense | 5' TGT GGT GAC GTA GCA ACG AGT TGC T 3' |
| NS5A Gen 1 in sense | 5' TCC GGT TCC TGG CTA AGR GA 3' |
| NS5A Gen 1 in antisense | 5' CAG GAG TAA GAC ATT GAG CAG CAC 3' |
| NS5A Gen 3 out sense | 5' CAC TAT GTT CCC GAG AGC G 3' |
| NS5A Gen 3 out antisense | 5' CGA AGG TAA CCT TCT TCT GAC G 3' |
| NS5A Gen 3 in sense | 5' GCG GTT ACA CCA GTG GAT CAA TG 3' |
| NS5A Gen 3 in antisense | 5' GTG TTA TCA TGG CGC CCG TCC 3' |
| NS5A Gen 5 out sense | 5' CAC TAC GTG CCC GAG ACG GAC GC 3' |
| NS5A Gen 5 out antisense | 5' CAA AAG TGA CCT TTT TCT GCC T 3' |
| NS5A Gen 5 in sense | 5' GAG GCT CCA CAC GTG GAT CGG TG 3' |
| NS5A Gen 5 in antisense | 5' GGG TGA TGA GCG CCC CCG TCC 3' |

**___________________________________________________________________________**

|  | **PCR first-round**  **primers** | **T°C hybridization** | **PCR second-round primers** | **T°C hybridization** |
| --- | --- | --- | --- | --- |
| **CORE Genotype 1/5** | CORE sense | 55 | none | none |
|  | CORE Gen 1/5 antisense |  | none |  |
| **CORE Genotype 3** | CORE sense | 55 | none | none |
|  | CORE Gen 3 antisense |  | none |  |
| **NS5A Genotype 1** | NS5A Gen 1 out sense | 49 or 55 | NS5A Gen 1 in sense | 49 or 55 |
|  | NS5A Gen 1 out antisense |  | NS5A Gen 1 in antisense |  |
| **NS5A Genotype 3** | NS5A Gen 3 out sense | 57 | NS5A Gen 3 in sense | 59 |
|  | NS5A Gen 3 out antisense |  | NS5A Gen 3 in antisense |  |
| **NS5A Genotype 5** | NS5A Gen 5 out sense | 46 | NS5A Gen 5 in sense | 57 |
|  | NS5A Gen 5 out antisense |  | NS5A Gen 5 in antisense |  |
